# Topological disruption of high‐order functional networks in cognitively preserved Parkinson's disease

**DOI:** 10.1111/cns.14037

**Published:** 2022-12-05

**Authors:** Song'an Shang, Siying Zhu, Jingtao Wu, Yao Xu, Lanlan Chen, Weiqiang Dou, Xindao Yin, Yu‐Chen Chen, Dejuan Shen, Jing Ye

**Affiliations:** ^1^ Department of Medical imaging center Clinical Medical College, Yangzhou University Yangzhou China; ^2^ Department of Neurology Clinical Medical College, Yangzhou University Yangzhou China; ^3^ MR Research China GE Healthcare Beijing China; ^4^ Department of Radiology Nanjing First Hospital, Nanjing Medical University Nanjing China

**Keywords:** functional connectivity, functional magnetic resonance imaging, graph theory, machine learning, Parkinson's disease

## Abstract

**Aims:**

This study aimed to characterize the topological alterations and classification performance of high‐order functional connectivity (HOFC) networks in cognitively preserved patients with Parkinson's disease (PD), relative to low‐order FC (LOFC) networks.

**Methods:**

The topological metrics of the constructed networks (LOFC and HOFC) obtained from fifty‐one cognitively normal patients with PD and 60 matched healthy control subjects were analyzed. The discriminative abilities were evaluated using machine learning approach.

**Results:**

The HOFC networks in the PD group showed decreased segregation and integration. The normalized clustering coefficient and small‐worldness in the HOFC networks were correlated to motor performance. The altered nodal centralities (distributed in the precuneus, putamen, lingual gyrus, supramarginal gyrus, motor area, postcentral gyrus and inferior occipital gyrus) and intermodular FC (frontoparietal and visual networks, sensorimotor and subcortical networks) were specific to HOFC networks. Several highly connected nodes (thalamus, paracentral lobule, calcarine fissure and precuneus) and improved classification performance were found based on HOFC profiles.

**Conclusion:**

This study identified disrupted topology of functional interactions at a high level with extensive alterations in topological properties and improved differentiation ability in patients with PD prior to clinical symptoms of cognitive impairment, providing complementary insights into complex neurodegeneration in PD.

## Graphical abstract

The present study investigated the topology of high‐order networks in cognitively normal patients with PD by employing HOFC construction. A correlation between network segregation and motor performance was observed in this study. Furthermore, we verified the greater sensitivity of HOFC profiles, in contrast to LOFC profiles, for the exploration of network disruptions prior to clinical evidence of cognitive impairment with more extensively altered topological properties and improved classification performance.

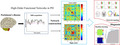

## 1 INTRODUCTION

Parkinson's disease (PD) is an aging‐related neurodegenerative disorder with marked movement disturbances (e.g., tremor, rigidity, bradykinesia and postural instability), which gradually deprives individuals of the ability to engage in motor activities and burdens quality of life.^1^ Given that PD is pathologically derived from dopaminergic dysfunction in the nigrostriatal pathways and progresses to heterogeneous impairments due to widespread involvement of the cerebral cortex, localizing aberrant alterations to particular brain regions in vivo is essential for early diagnosis and clinical intervention to retard progression.^2^ Functional MRI (fMRI) has been employed in neuroimaging studies as a robust, noninvasive approach for clinical diagnoses and the exploration of functional alterations prior to the structural changes in patients with PD, yielding convincing findings that are relevant to clinical symptoms and insights into the underlying pathological mechanisms.^3^, ^4^, ^5^

In contrast to changes in discrete regions, the notion that PD could be defined as disconnection syndrome has gained supporting evidence from a growing fMRI literature that has revealed abnormal functional connectivity (FC) within the cortico‐basal ganglia circuit and between disrupted brain networks, mainly the default mode network (DMN), sensorimotor network (SMN) and frontoparietal network (FPN).^6^, ^7^, ^8^ Subsequent studies have further highlighted the resultant alterations in network topology in individuals with PD on the basis of graph theory and characterized global, nodal and modular disruptions in these networks, which has provided insights into impaired information transmission from the perspective of the connectome.^9^, ^10^, ^11^ However, these documented topological abnormalities are less detectable in patients prior to clinical cognitive impairment, which is challenging for the comprehensive understanding of the role of disconnection and for timely diagnoses before the onset of cognitive dysfunction.

Recently, it has been reported that the constructed networks adopted in previous studies are traditionally based on the temporal correlation calculation between pairwise brain regions, which may not be sufficient for reflecting the complexity of functional interactions.^12^ In contrast to this low‐order FC (LOFC), high‐order FC (HOFC) was generated by forming pairwise functional coherence on the basis of LOFC profiles between regions.^13^ It has been validated that HOFC profiles could capture high‐level modulations in several pathological conditions (e.g., mild cognitive impairment, autism spectrum disorders, and episodic depression) with improved classification performance for diagnosis.^14^, ^15^, ^16^ Given that motor dysfunction in PD is not solely a disruption of motor‐related networks but a consequence of impaired high‐level cognitive processes,^17^ it is plausible that the high‐order information exchange in cognitively preserved patients with PD could be sensitively revealed in the topological properties of HOFC networks.

Considering that high‐order topological metrics, with a focus mainly on motor‐related alterations, remain poorly investigated, the present study sought to explore alterations of topological metrics via a graph‐theoretical approach on the basis of HOFC profiles and their relevance to clinical performance in cognitively normal patients with PD. Moreover, a machine learning approach was specifically adopted for the verification of classification performance of the fundamental HOFC between the enrolled patients and healthy controls (HCs). We hypothesized that the disrupted topology of HOFC networks might be more pronounced and achieve better discriminative ability than that of LOFC networks, providing a complementary understanding of the underlying mechanisms of motor deficits in those with PD.

## 2 MATERIALS AND METHODS

### 2.1 Patient cohort

From April 2020 to December 2021, fifty‐one right‐handed patients with a diagnosis of PD by two neurologists were recruited from the movement clinic. Additionally, sixty HCs matched by age, sex, handedness and years of education were also enrolled from the local communities during the same period. The ethics committee of Clinical Medical College, Yangzhou University approved this study protocol, and written informed consent was obtained from individuals 24 h before the clinical assessment. For patients with PD, the Unified Parkinson's Disease Rating Scale part III (UPDRS‐III) and Hoehn and Yahr (H‐Y) scale were applied to evaluate disease severity and stage, respectively. The Mini‐Mental State Examination (MMSE) and Montreal Cognitive Assessment (MoCA) were completed by all participants for the assessment of global cognition. During the daytime (8:00 a.m. to 18:00 p.m.), clinical assessments were performed by the above two neurologists before the MRI data acquisition, and patients with PD were instructed to withdraw antiparkinsonian medications for more than 12 h.

The inclusion criteria for patients with PD were the fulfillment of Level II of the recommended Movement Disorder Society (MDS) criteria,^18^ early‐to‐mid stage with H‐Y stage of I‐II and stable antiparkinsonian medications for at least 4 weeks. Subjects were excluded if they met any of the following criteria: (1) family history of PD, (2) presence of other psychiatric or neurological disorders (e.g. Alzheimer's disease, schizophrenia, depression, and epilepsy), (3) history of vascular diseases, trauma, lesions or complications that involved the central nervous system, (4) clinically impaired global cognition with MMSE score < 26 and MoCA score ≤ 26, (5) alcohol or substance abuse, (6) any MRI contradictions or severe visual or auditory impairment that would prevent scanning, and (7) poor image quality caused by field distortions, head motion or intensity inhomogeneity.

### 2.2 MRI acquisition

The MRI data of each subject were acquired using an MRI scanner (3.0‐tesla Discovery MR750; GE Medical Systems) with an eight‐channel phased array head coil. Each subject lay supinely with head fixation by foam pads and hearing protection by earplugs. All the participants were instructed to stay awake with their eyes closed and to avoid any deliberate thoughts. The image quality and status of each individual were monitored during scanning, and a report of whether they fell asleep was also obtained. Functional images were acquired using a gradient recalled echo echo‐planar imaging sequence with the following parameters: repetition time (TR): 2000 ms, echo time (TE): 30 ms, flip angle: 90°, slice thickness: 4 mm without gaps, field of view (FOV): 240 × 240 mm^2^, matrix size: 64 × 64, voxel size: 4.0 × 4.0 × 4.0 mm^3^, and number of time points: 240. Each frame included 35 continuous slices that covered the whole brain volume. The slices were parallel to the anterior/posterior commissure, and the total scan time was 8 min and 30 s. Structural data (high‐resolution T1‐weighted images) for registration were also obtained using a 3D whole‐brain brain volume imaging sequence with the following parameters: TR: 12 ms, TE: 5.1 ms, inversion time: 450 ms, flip angle: 15°, slice thickness: 1 mm without gaps, FOV: 240 × 240 mm^2^, matrix size: 256 × 256, voxel size: 1 × 1 × 1 mm^3^, number of slices: 172, and total scan time: 5 min and 20 s.

### 2.3 Data preprocessing

The functional data were preprocessed using the Data Processing and Analysis of Brain Imaging toolbox (DPABI, Version 4.5, http://rfmri.org/dpabi) based on Statistical Parametric Mapping (SPM, version 12, http://www.fil.ion.ucl.ac.uk/spm) in MATLAB software (version 2018a, MathWorks) according to routine procedures as previously described.^19^, ^20^ Briefly, the procedures included the following steps: removal of the first 10 volumes, slice timing correction, realignment for head motion by eliminating subjects with displacement >2 mm or rotation >2.0 ,^15^ spatial normalization to Montreal Neurological Institute space at a resampling of 3 mm × 3 mm × 3 mm by their corresponding structural data, detrending and bandpass filtering (0.01–0.08 Hz), nuisance signal regression (including white matter and cerebrospinal fluid signals and the Friston‐24 parameters). To minimize the spurious functional connectivity introduced by spatial smoothing and global signal regression, we did not conduct these steps with reference to previous literature.^21^, ^22^ The mean framewise displacement (FD) values for each subject was also generated as metric of head motion.

### 2.4 Network construction and topological properties

The construction of the networks (LOFC and HOFC), composed of nodes and edges, was performed using the GRETNA toolbox (version 2.0, http://www.nitrc.org/projects/gretna/). The nodes of both LOFC and HOFC networks were defined by brain regions that were segmented by the automated anatomic labeling (AAL) atlas (2nd version),^23^ resulting in 90 nodes for each subject. Regarding edge definition, Pearson's correlation coefficient of the mean time series (LOFC‐network) or that of the LOFC profiles (HOFC‐network) between any two nodes was calculated using the BrainNetClass toolbox (version 1.1, https://github.com/zzstefan/BrainNetClass). A set of functional matrices (90 × 90) were built and subsequently transformed to binary matrices (removal of negative connection coefficients) with a given network sparsity. The network sparsity threshold was evaluated across a wide range from 10% to 50% at a step of 0.01, ensuring the unbiased intergroup differences in network organization.^10^, ^24^ The topological properties for individuals were calculated at each sparsity level and were further compared between groups using the values of the area under the curve (AUC) over the sparsity range. Briefly, the topological properties included global metrics of normalized clustering coefficient (γ), normalized characteristic path length (*λ*), small‐worldness (*σ*), characteristic path length (Lp), clustering coefficient (Cp), global efficiency (Eglob) and local efficiency (Eloc); the nodal metrics included nodal degree, nodal efficiency, and nodal betweenness; and modular architecture consisted of intra‐ or intermodular connectivity within 5 modules of SMN, DMN, FPN, visual network (VN) and subcortical network (SN).^11^

### 2.5 Statistical analysis

Kolmogorov–Smirnov tests were utilized to check the normality of the clinical data and topological metric distributions. The comparisons of age, FD and sex between the PD and HC groups were performed by independent‐samples *t* test and chi‐square (*χ* ^2^) tests, respectively. The intergroup differences in education (years) and clinical assessments (scores) were analyzed using Mann–Whitney *U* tests. The above statistical analyses were conducted using SPSS software (version 19.0; SPSS Inc.), and the threshold for statistical significance was set to a *p* value <0.05.

The AUC values of topological properties were compared between groups using a two‐sample *t* test in the GRETNA toolbox. A *p* value <0.05 was defined as statistically significant. A false discovery rate (FDR) at a threshold of *p* < 0.05 was adopted for the multiple comparison correction of nodal metrics. The network‐based statistic (NBS) approach in the GRETNA toolbox was utilized for identification of intergroup differences in FC between paired brain regions at a threshold of *p* < 0.05 with corrected *p* < 0.001 (10,000 permutations). The exploration of potential correlations between topological metrics and clinical scale scores in the PD group was conducted using the Spearman rank correlation test with the SPSS 19.0 software at a statistically significant threshold of *p* value <0.05. The covariates for the above analyses included age, sex and education (years).

For both LOFC and HOFC networks, we employed the connection coefficient of each node as a feature vector and utilized the least absolute shrinkage and selection operator (LASSO) at lambda = 0.05 for feature selection, improving the classification performance and model robustness. Considering the relatively small sample size in the present study, we applied leave‐one‐out cross‐validation (LOOCV), with superior performance to minimize the possibility of overfitting,^25^, ^26^ to optimize the parameters and evaluate the model. The support vector machine approach was adopted to perform the classification, including a determination of the AUC of the receiver operator characteristic curve with accuracy, sensitivity, and specificity generated for the depiction of classification performance.

### 2.6 Validation analysis

Given the diverse strategies for functional network definition, we validated the classification performance of functional connectivity and the analysis of topological properties by applying another widely used atlas (455 ROIs for node definition), including Schaefer functional atlas (400 ROIs responding to 7 networks)^27^ and subcortical functional atlas (54 ROIs composing of SN).^28^

## 3 RESULTS

### 3.1 Demographic and clinical variables

The HC group was demographically matched to the PD group in terms of age, distribution of sex and years of education (*p* > 0.05, respectively). There was no significant intergroup difference of mean FD (*p* > 0.05). The patients with PD exhibited movement disorder, which was assessed using the UPDRS‐III scale, with a mean score of 28.88 ± 15.27 and were at an early‐mid stage, which was evaluated by the H‐Y scale, with a mean score of 1.47 ± 0.50. Additionally, global cognition in both groups was normal, with no significant intergroup difference in MMSE scores and MoCA scores (*p* > 0.05, respectively). The demographic information and clinical assessments for all participants are summarized in Table 1.

**TABLE 1:** Demographic information and scores of clinical scales.

	HC (*n* = 60)	PD (*n* = 51)	T/Z	*p*
Age	61.63 ± 6.39	60.69 ± 11.71	0.54	0.59
Sex (M/F)	35/25	31/20	0.069	0.73
Education (years)	12.60 ± 2.50	12.08 ± 2.63	−1.13	0.26
Disease duration (years)	N/A	3.43 ± 2.12	N/A	N/A
UPDRS‐III	N/A	28.88 ± 15.27	N/A	N/A
H‐Y stage	N/A	1.47 ± 0.50	N/A	N/A
MMSE	28.97 ± 1.02	28.63 ± 1.01	−1.77	0.08
MoCA	27.77 ± 1.36	27.24 ± 1.18	−1.91	0.06
FD (mm)	0.09 ± 0.02	0.09 ± 0.01	−0.45	0.65

*Note*: Data was represented as mean ± SD; N/A indicates not applicable.

Abbreviations: F, female; FD, Framewise displacement; HC, healthy control; H‐Y, Hoehn‐Yahr; M, male; MMSE, Mini‐mental State Examination; MoCA, Montreal Cognitive Assessment; PD, Parkinson's disease; UPDRS, Unified Parkinson's Disease Rating Scale.

### 3.2 Aberrant global topologic metrics of constructed networks

**FIGURE 1:**
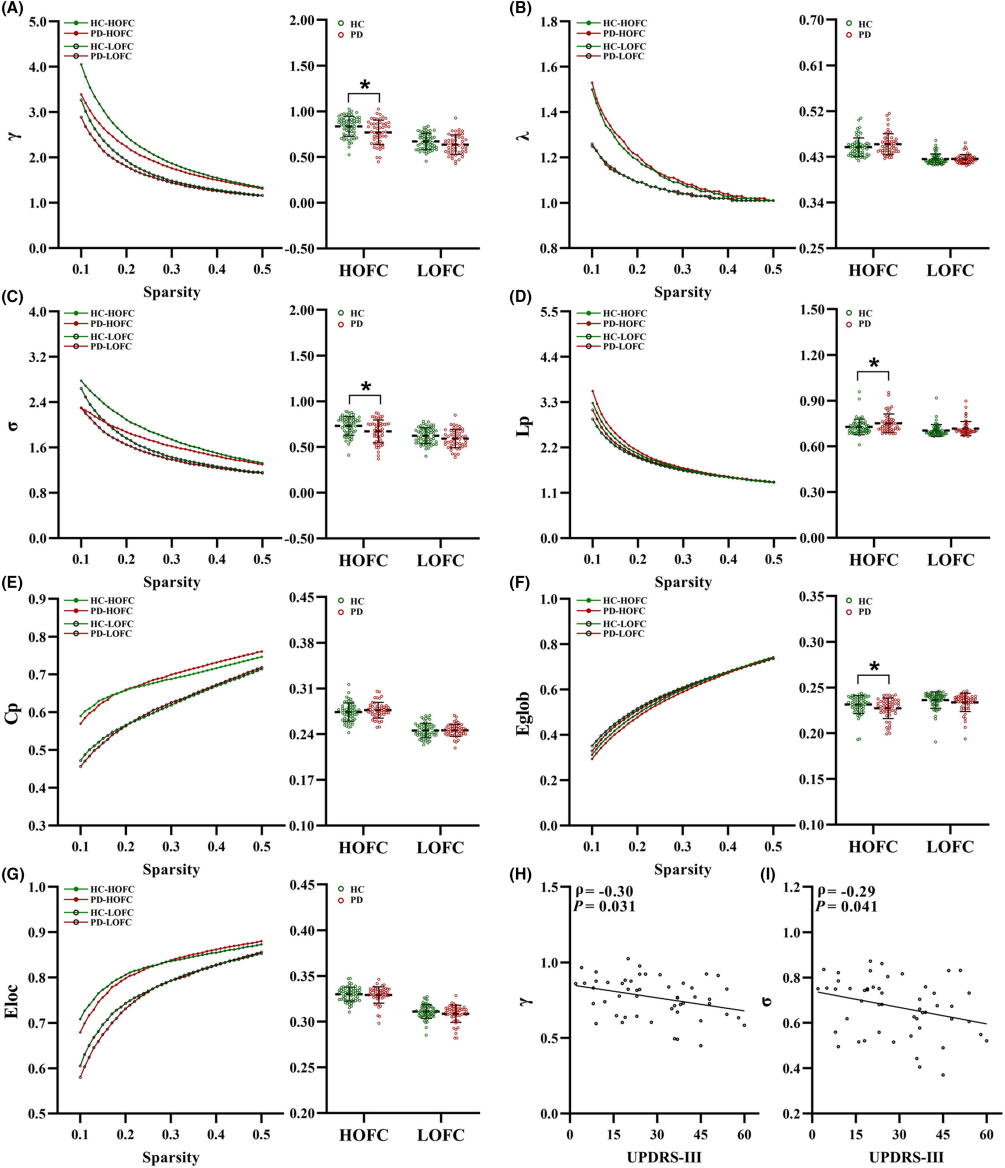
Network topology of global properties, intergroup comparisons and correlations to clinical assessments. The global metrics include *γ* (A) *λ* (B) *σ* (C) Lp (D) Cp (E) Eglob (F) and Eloc (G). Significant intergroup differences were observed in HOFC‐networks with metrics of *γ*, *σ*, Eglob and Lp (*p* < 0.05, respectively). Scatterplots showing linkage between UPDRS‐III scores and values of *γ* (H) and σ (I) for HOFC‐networks in PD group. * indicates significant intergroup difference with *p* < 0.05. Cp, clustering coefficient; Eglob, global efficiency; Eloc, local efficiency; HC, healthy control; HOFC, high‐order functional connectivity; LOFC, low‐order functional connectivity; Lp, characteristic path length; PD, Parkinson's disease; UPDRS, Unified Parkinson's disease rating scale; *γ*, normalized clustering coefficient; *λ*, normalized characteristic path length; *σ*, small‐worldness.

The network topology of both groups at the global level is shown in Figure 1A–G. Across the network sparsity thresholds, both the PD and HC groups demonstrated small‐world topological organization with *γ* > 1 and *λ* ≈ 1 regardless of the constructions of functional networks (LOFC and HOFC). For HOFC networks, significantly lower *γ*, *σ*, and Eglob values but higher Lp values were observed in patients with PD than in HC subjects (*p* < 0.05, respectively) (Figure 1A,C,D,F). However, there were no significant intergroup differences in any global properties in the LOFC networks (*p* > 0.05, respectively). The detailed global metrics for each group are listed in Table S1. For the HOFC networks in the PD group, we also found that disease severity scored by the UPDRS‐III scale was significantly correlated with disrupted global properties of *γ* (*ρ* = −0.30, *p* = 0.031) and *σ* (*ρ* = −0.29, *p* = 0.041) (Figure 1H,I).

### 3.3 Disturbed nodal metrics of functional connectome

**FIGURE 2:**
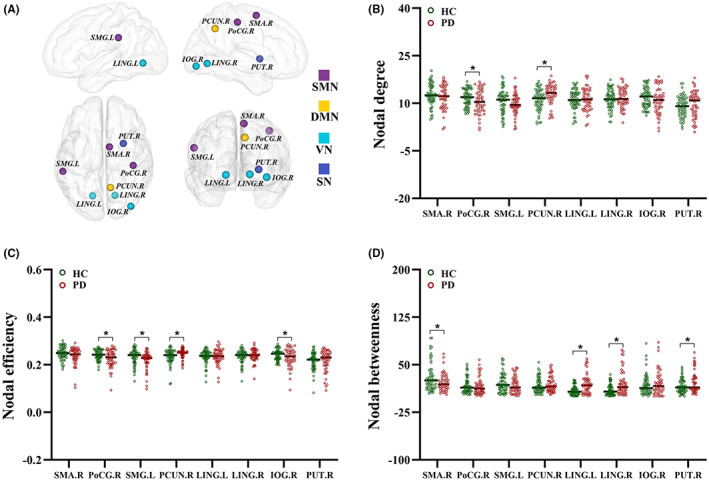
Specific brain regions with altered nodal metrics in HOFC‐networks. The nodes with significant intergroup difference of nodal centralities were distributed in SMN, DMN, VN and SN (A) (*p* < 0.05, FDR corrected, respectively). The nodal centralities include nodal degree (B) nodal efficiency (C) and nodal betweenness (D). * indicates significant intergroup difference with *p* < 0.05. DMN, default mode network; FDR, false discovery rate; HC, healthy control; HOFC, high‐order functional connectivity; IOG, inferior occipital gyrus; L, left; LING, lingual gyrus; LOFC, low‐order functional connectivity; PCUN, precuneus; PD, Parkinson's disease; PoCG, postcentral gyrus; PUT, putamen; R, right; SMA, supplementary motor area; SMG, supramarginal gyrus; SMN, sensorimotor network; SN, subcortical network; VN, visual network.

The nodes with significant intergroup differences (at least one metric) in the HOFC networks are summarized in Table S2. Nodal centralities in the HOFC networks in the PD group were significantly higher in the DMN, FPN, VN and SN but lower in the SMN, DMN, VN and SN (*p* < 0.05, FDR corrected, respectively) than in the HC group. Notably, despite the overlapping regions with those of the LOFC networks, as listed in Table S3, we observed that several regions (Figure 2A), including the precuneus, putamen, lingual gyrus, supramarginal gyrus, supplementary motor area, postcentral gyrus and inferior occipital gyrus, featured altered nodal metrics that were specific to HOFC networks (Figure 2B–D).

### 3.4 Altered modular architecture and connections of topological organization

**FIGURE 3:**
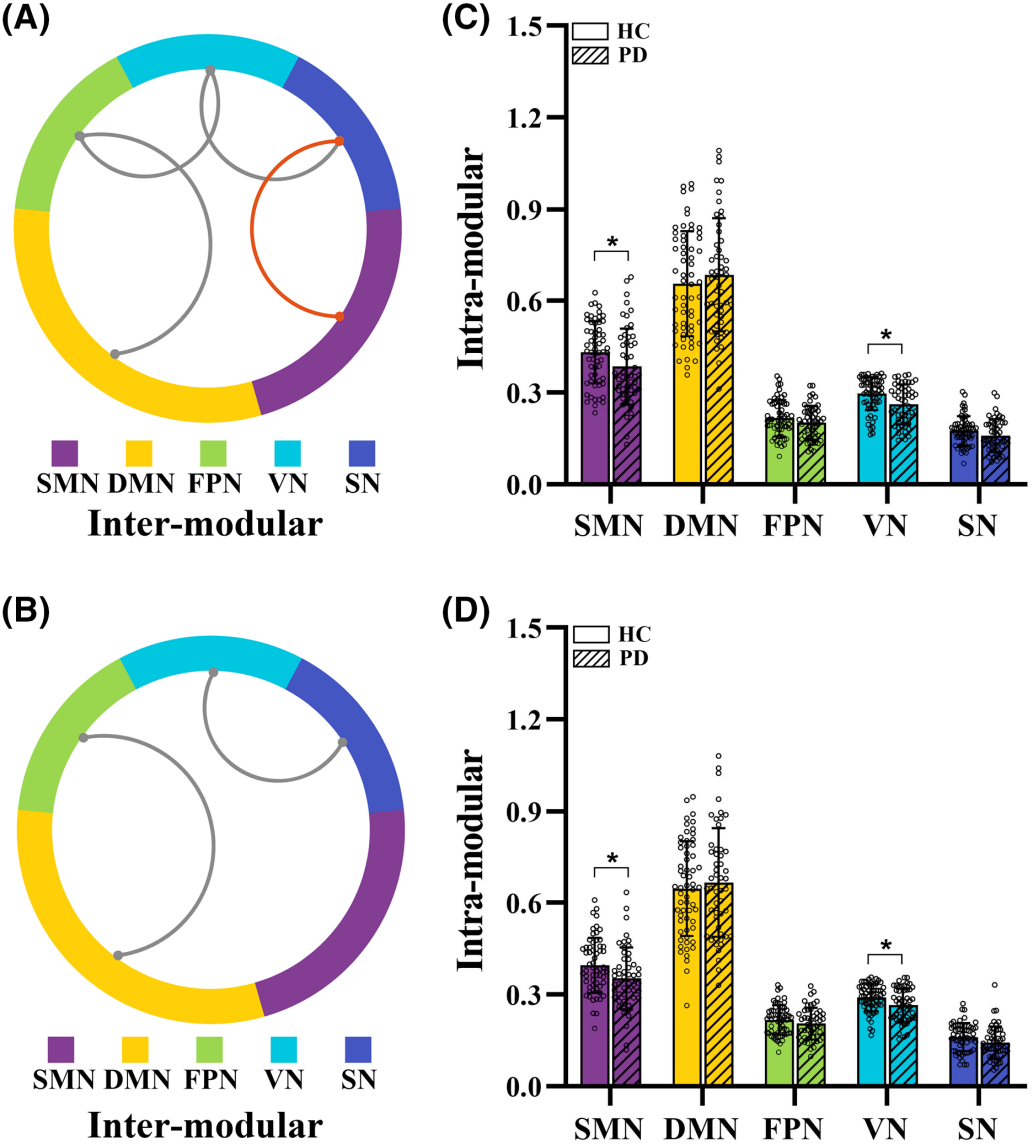
Aberrant modular architectures of functional networks for each group. Significantly altered inter‐modular functional connectivity between groups was observed in HOFC‐networks (A) and LOFC‐networks (B) (*p* < 0.05, respectively). Red curve indicates significantly higher values in PD group than that of HC group, and gray curve indicates significantly lower values in PD group than that of HC group. Significantly altered intra‐modular functional connectivity between groups was observed in HOFC‐networks (C) and LOFC‐networks (D) (*p* < 0.05, respectively). * indicates significant intergroup difference with *p* < 0.05. DMN, default mode network; FPN, fronto‐parietal network; HC, healthy control; HOFC, high‐order functional connectivity; LOFC, low‐order functional connectivity; PD, Parkinson's disease; SMN, sensorimotor network; SN, subcortical network; VN, visual network.

For the topological analysis of modular architecture based on HOFC or LOFC, we both found significantly greater intermodular FC between the DMN and FPN and the VN and SN, as well as significantly less intramodular FC within the SMN and VN in the PD group than in the HC group (*p* < 0.05, respectively) (Figure 3A,B). Moreover, significantly higher intermodular FC between the FPN and VN and lower FC between the SMN and SN were observed in patients with PD than in HC subjects with the HOFC construction (*p* < 0.05, respectively) (Figure 3A). For both HOFC and LOFC networks, the intramodular FC in the SMN and VN was significantly lower in the PD group than in the HC group (*p* < 0.05, respectively) (Figure 3C,D). The detailed modular metrics for each group are listed in Table S4.

**FIGURE 4:**
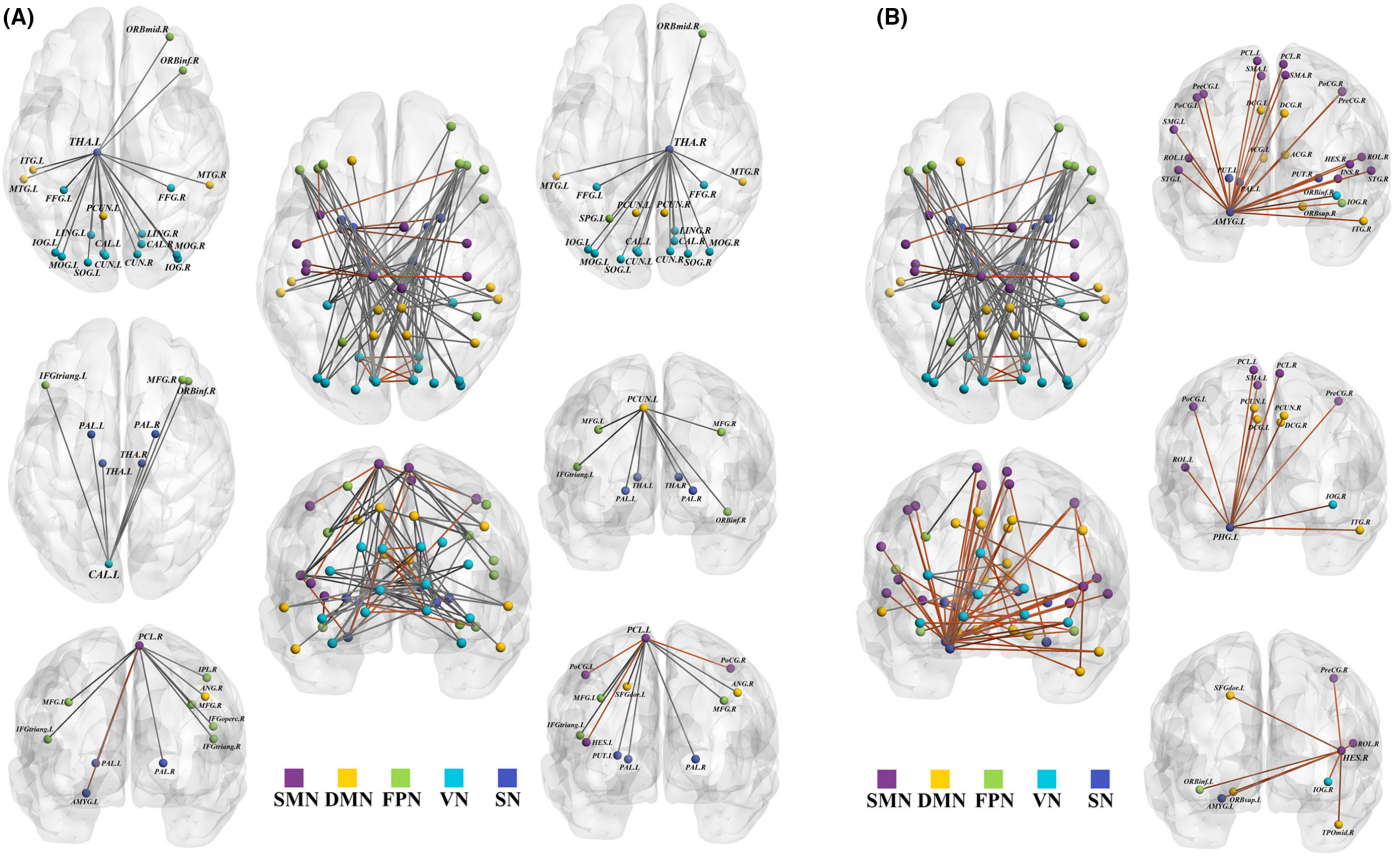
Altered functional connections of functional networks for each group. NBS analysis revealed significant altered functional connections featured highly connected nodes in HOFC‐networks (A) and LOFC‐networks (B) (*p* < 0.05, NBS corrected). Red line indicates significantly higher values in PD group than that of HC group, and gray line indicates significantly lower values in PD group than that of HC group. ACG, anterior cingulate gyrus; AMYG, amygdala; ANG, angular gyrus; CAL, calcarine fissure; CUN, cuneus; DCG, median cingulate gyrus; DMN, default mode network; FFG, fusiform gyrus; FPN, fronto‐parietal network; HC, healthy control; HES, heschl gyrus; HOFC, high‐order functional connectivity; IFGoperc, inferior frontal gyrus, opercular part; IFGtriang, inferior frontal gyrus, triangular part; INS, insula; IOG, inferior occipital gyrus; IPL, inferior parietal gyrus; ITG, inferior temporal gyrus; L, left; LING, lingual gyrus; LOFC, low‐order functional connectivity; MFG, middle frontal gyrus; MOG, middle occipital gyrus; MTG, middle temporal gyrus; NBS, Network‐based statistic; ORBinf, inferior frontal gyrus, orbital part; ORBmid, middle frontal gyrus, orbital part; ORBsup, superior frontal gyrus, orbital part; ORBsupmed, superior frontal gyrus, medial orbital; PAL, pallidum; PCL, paracentral lobule; PCUN, precuneus; PD, Parkinson's disease; PHG, parahippocampal gyrus; PoCG, postcentral gyrus; PreCG, precental gyrus; PUT, putamen; R, right; ROL, rolandic operculum; SFGdor, superior frontal gyrus, dorsolateral; SMA, motor area; SMG, supramarginal gyrus; SMN, sensorimotor network; SN, subcortical network;SOG, superior occipital gyrus; SPG, superior parietal gyrus; STG, superior temporal gyrus; THA, thalamus; TPOmid, temporal pole: middle temporal gyrus; VN, visual network.

We identified 96 connections and 50 nodes in HOFC networks and 70 connections and 54 nodes in LOFC networks that were significantly different between groups (*p* < 0.05, NBS corrected). The nodes with more than 5 connections in the HOFC networks included the right thalamus, left thalamus, left paracentral lobule, right paracentral lobule, left calcarine fissure and left precuneus, which was more than the number of such nodes in the LOFC networks, which included the left amygdala, left parahippocampal gyrus and right heschl gyrus (Figure 4A,B).

### 3.5 Classification performance of functional connectivity

**FIGURE 5:**
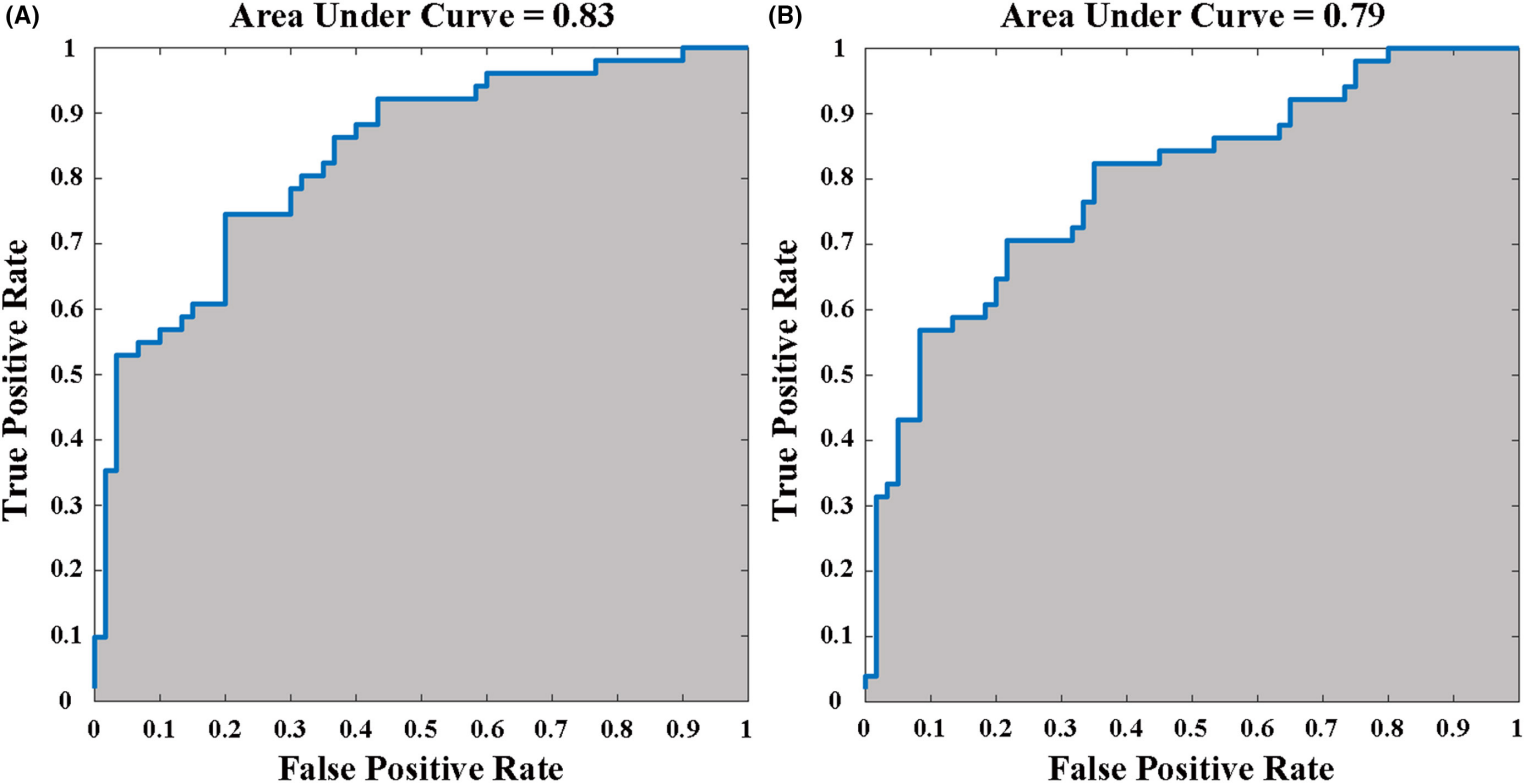
Classification performances of functional networks. The classification performance of HOFC‐networks based on functional connectivity features (A; AUC 0.83, accuracy 75.68%, sensitivity 74.51% and specificity 76.67%) was improved than that of LOFC‐networks (B; AUC 0.79, accuracy 73.87%, sensitivity 70.59% and specificity 76.67%). AUC, area under the curve; HOFC, high‐order functional connectivity; LOFC, low‐order functional connectivity.

The features based on HOFC profiles achieved an AUC of 0.83, with an accuracy of 75.68%, sensitivity of 74.51% and specificity of 76.67%, for the differentiation between the PD and HC groups (Figure 5A). Meanwhile, the classification performance of connection features in the LOFC network had an AUC of 0.79, with an accuracy of 73.87%, sensitivity of 70.59% and specificity of 76.67%, which was lower than that with the HOFC (Figure 5B).

### 3.6 Reproducibility

The main findings were largely reproducible when utilizing different strategy for network parcellation (Tables S5–S8).

## 4 DISCUSSION

The present study investigated the topology of high‐order networks in cognitively normal patients with PD by employing HOFC construction. Relative to those of matched HC subjects, high‐level information interactions in these patients were disrupted and showed altered global properties of decreased segregation (lower values of Eglob, γ, and σ) and integration (higher values of Lp), aberrant nodal metrics that were mainly distributed in the SMN, DMN, FPN, VN and SN, and impaired pairwise connections and modular architecture. A correlation between network segregation and motor performance was also observed in this study. Furthermore, we verified the greater sensitivity of HOFC profiles, in contrast to LOFC profiles, for the exploration of network disruptions prior to clinical evidence of cognitive impairment with more extensively altered topological properties and improved classification performance.

The weakened small‐world characteristics derived from LOFC profiles in patients with PD have been well documented in previous studies using graph‐theoretical approaches.^9^, ^10^, ^11^ Nevertheless, cognitively preserved patients with PD have subtle changes with decreased segregation or integration of the functional network, which are more detectable in cognitively impaired patients with PD.^10^, ^24^ Our findings also failed to reveal any significant intergroup differences in global topographic properties based on LOFC. In this regard, the traditional construction of functional networks might not allow the exploration of motor‐related information exchange in PD. Benefiting from the sensitivity of HOFC for the modeling of high‐level functional interactions,^29^ the present study found that the HOFC network in the HC group exhibited small‐world properties, indicating that functional interactions at high levels still maintain an efficient network for the balance between local specialization and global integration, which was in accordance with that of traditional functional networks.^24^ Particularly, the PD group exhibited with decreased Eglob, γ and σ values relative to those of HC group, indicating a shift toward random organizations rather the preservation of small‐worldness. Taking into their links to disease severity, we could confirm the association of impaired functional networks at high levels with motor‐related neurodegeneration prior to clinical cognitive impairment.

The impaired information communication at the global level might be a consequence of disturbed regional properties within particular regions. In addition to the findings that the distribution of altered nodes within HOFC networks was more extensive than that of LOFC networks, we observed that several regions were specifically disrupted in the high‐order interactions. Decreased nodal centralities in the PD group were found in three regions, the key components of the SMN that are responsible for sensory integration and motor initiation.^30^, ^31^ This pronounced finding was in accordance with decreased regional activities^32^ and FC^33^ in the SMN, which reinforces the viewpoint that the SMN is preferentially involved as a vulnerable functional unit in the early stages of PD before the appearance of clinically impaired cognition.^34^ We also discovered that increased nodal centralities in patients with PD were located in the putamen, which is crucial for movement coordination attributed to dopaminergic dysfunction,^35^ as well as in the nodes that compose the VN or DMN. Several studies have indicated that disturbances in the VN are the major complex sensory alterations in PD that lead to aberrant high‐order processing (visual hallucinations and perceptual and cognitive dysfunction) prior to clinical evidence.^36^, ^37^ Meanwhile, the DMN is the well‐defined core network implicated in high‐level cognitive processes, and its relevance to motor disturbance or cognitive impairment in PD has been documented.^38^, ^39^ Collectively, these nodal alterations of HOFC networks reinforced the understanding of complex high‐level information processing related to movement disorders in PD patients with normal cognition.

Additionally, we analyzed the intergroup differences in edge strength for the exploration of altered internetwork associations and information flow in the PD group, which could be explicitly captured via HOFC by measuring the pairwise LOFC profiles rather than the fundamental time series between any two regions.^13^ Some meaningful nodes (thalamus, paracentral lobule, calcarine fissure and precuneus) with higher interactions were observed in cognitively normal patients with PD, in addition to the extensively aberrant connections between HOFC networks. These prominent regions were notably found to be vulnerable targets with disrupted connections to other nodes in different networks in the current study, which was beyond their roles as hubs within individual networks (SN, SMN, VN and DMN).^24^, ^40^ These findings could be interpreted by the complexity of the brain connectome that consists of both low‐order acquisitions from each region and high‐order modulation via internetwork interactions, which turned out to be sensitive to disruption in the pathological status.^16^, ^41^ Because of this, we also discovered modular abnormalities in the HOFC constructed networks with decreased intramodular strength (e.g., in the SMN and VN) as well as intermodular information flow between the SMN and SN, both of which are associated with movement disorders, but increased intermodular functional connectivity in cortico‐basal ganglia circuit and cognition‐related networks that might be a potential compensatory response in the early stage of PD. However, the precise interpretability for these metrics based on HOFC profiles warrants further investigation.

Although many studies have concentrated on the intergroup differences in topological properties based on traditional network construction, it is urgent to identify the classification performance of these potential imaging biomarkers in PD for individual clinical diagnosis.^42^, ^43^ Given that the connectome‐wide FC profiles could achieve higher diagnostic value than that of graph‐derived metrics,^44^, ^45^ we subsequently observed that HOFC features rather than LOFC features improved the diagnostic capability to differentiate between groups of PD patients and HCs by utilizing a robust machine learning approach with LASSO and SVM. However, we note that the power of HOFC for PD classification was not as prominent as previous studies with a focus on patients with cognitive impairment.^12^, ^16^, ^46^ Several longitudinal studies have proven that functional abnormalities in PD progressively worsen or spread from normal cognition to mild cognitive impairment and even dementia but have emphasized the importance of those early alterations prior to clinical symptoms.^47^, ^48^ Hence, the aberrant topological architecture in HOFC at least provided insights into high‐order functional interactions in cognitively unimpaired patients with PD and primarily validated the assumption of HOFC for efficient imaging biomarker detection. In the future, more works with machine learning should be engaged in the validation of HOFC in other datasets (e.g., patients with PD from other institutions) or publicly available datasets (e.g., Parkinson progression marker initiative).

There are several limitations that should be recognized in the current study. First, the statistical power of our findings needs further validation in a larger cohort in the future, which might have been influenced by the relatively small sample size. Second, global cognitive performance was evaluated by the MMSE and MoCA, whereas a battery of cognitive assessments would provide greater benefit the exploration of clinical relevance related to cognitive alterations. Third, although several pronounced results were found using HOFC, a more refined delineation of the corresponding mechanisms deserves attention at the fundamental level of research, as well as urgent conversions for clinical application (e.g., early diagnosis, differentiations among subtypes). Fourth, we minimized the effect of dopaminergic medications by instructing patients to withdraw from their antiparkinsonian treatment before the clinical assessments and MRI acquisition. However, enrollment of drug‐native patients with PD would be optimal for ruling out this confounding concern. Finally, the AAL atlas was employed for node definition, and HOFC was assessed as a static pattern of neuronal activity in this preliminary study. The performance of dynamic HOFC profiles using different parcellation strategies needs to be evaluated in future studies.

In conclusion, the current study revealed the topology of HOFC networks in cognitively preserved patients with PD. The enrolled patients exhibited disrupted high‐order information exchanges with lower small‐worldness and extensively altered nodal centralities. The high‐level functional interactions among networks featured aberrant hubs within the networks and disrupted information flow between the network modules. These findings supplied empirical evidence for comprehensive insights into the neurodegeneration associated with PD from the viewpoint of the functional connectome at a higher order and highlighted the promising prospect of HOFC profiles for the exploration of high‐level functional processes that underlie pathological status.

## FUNDING INFORMATION

This work was supported by the National Natural Science Foundation of China (82202120); Natural Science Foundation of Jiangsu Province (BK20201118, BK20201220); Jiangsu Provincial Special Program of Medical Science (BE2021604); Medical Research Project of Jiangsu Provincial Health Commission (M2022012); Science and Technology of Yangzhou (YZ2022071).

## CONFLICT OF INTEREST

The authors have declared that no conflict of interest exists.

## Supporting information


Tables S1‐S8

## Data Availability

The data that support the findings of present study are available from the corresponding author through reasonable request.
