# Supplementary material for: Topological disruption of high‐order functional networks in cognitively preserved Parkinson's disease
Source: CNS Neurosci Ther. 2022 Dec 5;29(2):566–76. doi: 10.1111/cns.14037 (PMC9873517; doi:10.1111/cns.14037)
Supplement: Supplementary file 1 — Tables S1‐S8 [file CNS-29-566-s001.docx]

Supplementary Table 1. Global topological metrics of HOFC-networks and LOFC-networks for each group

| Metric | HOFC | | | |  | LOFC | | | |
| --- | --- | --- | --- | --- | --- | --- | --- | --- | --- |
|  | HC | PD | T | *P* |  | HC | PD | T | *P* |
| γ | 0.84±0.11 | 0.77±0.13 | 2.89 | 0.005 |  | 0.67±0.09 | 0.64±0.11 | 1.89 | 0.06 |
| λ | 0.45±0.02 | 0.45±0.02 | -1.19 | 0.24 |  | 0.43±0.01 | 0.43±0.01 | -0.17 | 0.87 |
| σ | 0.73±0.10 | 0.67±0.13 | 2.83 | 0.006 |  | 0.62±0.09 | 0.59±0.10 | 1.80 | 0.07 |
| Lp | 0.73±0.05 | 0.75±0.06 | -2.25 | 0.03 |  | 0.70±0.04 | 0.72±0.05 | -1.55 | 0.12 |
| Cp | 0.27±0.01 | 0.28±0.01 | -1.07 | 0.29 |  | 0.25±0.01 | 0.25±0.01 | -0.10 | 0.92 |
| Eglob | 0.23±0.01 | 0.22±0.01 | 2.04 | 0.04 |  | 0.24±0.01 | 0.23±0.01 | 1.38 | 0.17 |
| Eloc | 0.33±0.01 | 0.33±0.01 | 0.54 | 0.59 |  | 0.31±0.01 | 0.31±0.01 | 1.56 | 0.12 |

Data was represented as mean ± standard deviation

Abbreviations: HC, healthy control; PD, Parkinson’s disease; HOFC, high-order functional connectivity; LOFC, low-order functional connectivity; γ, normalized clustering coefficient; λ, normalized characteristic path length; σ, small-worldness; Lp, characteristic path length; Cp, clustering coefficient; Eglob, global efﬁciency; Eloc, local efﬁciency

Supplementary Table 2. Nodal topological metrics of HOFC-networks for each group

| Brain region | Nodal degree | | | |  | Nodal efficiency | | | |  | Nodal betweenness | | | |
| --- | --- | --- | --- | --- | --- | --- | --- | --- | --- | --- | --- | --- | --- | --- |
|  | HC | PD | T | *P* |  | HC | PD | T | *P* |  | HC | PD | T | *P* |
| PCG.R | 8.99±3.86 | 11.36±4.00 | -3.15 | 0.002 |  | 0.21±0.04 | 0.23±0.03 | -3.21 | 0.002 |  | 14.21±17.22 | 14.19±8.65 | 0.001 | 0.99 |
| ANG.R | 9.17±3.42 | 11.65±3.51 | -3.73 | <0.001 |  | 0.22±0.03 | 0.24±0.03 | -3.29 | 0.001 |  | 14.94±15.57 | 20.56±23.84 | -1.48 | 0.14 |
| PCUN.R | 11.04±3.26 | 12.85±3.13 | -2.93 | 0.004 |  | 0.23±0.03 | 0.25±0.02 | -2.44 | 0.02 |  | 20.59±23.04 | 19.4±12.14 | 0.33 | 0.74 |
| CAL.R | 10.47±2.96 | 11.95±3.17 | -2.53 | 0.01 |  | 0.23±0.03 | 0.24±0.03 | -2.01 | 0.04 |  | 7.96±7.60 | 16.7±17.40 | -3.49 | 0.001 |
| LING.L | 11.15±2.96 | 11.21±3.45 | -0.10 | 0.92 |  | 0.23±0.03 | 0.24±0.03 | -0.17 | 0.87 |  | 11.45±18.62 | 21.13±19.90 | -3.62 | 0.001 |
| LING.R | 11.28±2.91 | 11.77±3.01 | -0.85 | 0.40 |  | 0.24±0.03 | 0.24±0.03 | -0.81 | 0.42 |  | 10.30±8.53 | 21.87±21.04 | -3.87 | <0.001 |
| PUT.R | 8.98±3.18 | 9.67±4.01 | -1.01 | 0.32 |  | 0.22±0.03 | 0.22±0.04 | -0.07 | 0.95 |  | 16.32±10.86 | 22.30±17.04 | -3.22 | 0.003 |
| THA.L | 8.66±4.05 | 11.90±3.94 | -4.23 | <0.001 |  | 0.21±0.05 | 0.24±0.03 | -3.83 | <0.001 |  | 15.30±13.96 | 24.90±17.97 | -3.14 | 0.002 |
| THA.R | 8.75±4.02 | 11.57±4.12 | -3.61 | <0.001 |  | 0.21±0.05 | 0.24±0.03 | -3.41 | 0.001 |  | 18.19±19.37 | 25.75±19.51 | -2.03 | 0.04 |
| ROL.L | 11.41±3.31 | 9.40±3.24 | 3.19 | 0.002 |  | 0.24±0.03 | 0.22±0.04 | 3.23 | 0.002 |  | 20.27±12.82 | 23.80±29.07 | -0.84 | 0.40 |
| ROL.R | 11.31±3.30 | 9.30±3.89 | 2.93 | 0.004 |  | 0.24±0.03 | 0.21±0.04 | 3.07 | 0.003 |  | 23.25±23.81 | 19.49±17.35 | 0.93 | 0.36 |
| SMA.R | 12.67±3.38 | 11.90±3.65 | 1.14 | 0.26 |  | 0.25±0.03 | 0.24±0.04 | 2.98 | 0.005 |  | 32.25±25.16 | 23.64±18.79 | 1.99 | 0.04 |
| PoCG.R | 11.77±2.64 | 10.46±3.71 | 2.95 | 0.003 |  | 0.24±0.02 | 0.23±0.04 | 2.62 | 0.01 |  | 18.74±17.97 | 16.61±15.85 | 0.65 | 0.52 |
| SMG.L | 10.72±3.64 | 9.69±3.25 | 1.54 | 0.13 |  | 0.23±0.03 | 0.22±0.04 | 3.08 | 0.003 |  | 25.14±24.57 | 17.8±12.77 | 1.91 | 0.06 |
| HES.L | 9.56±3.13 | 7.73±4.21 | 2.60 | 0.01 |  | 0.22±0.03 | 0.19±0.06 | 3.41 | 0.001 |  | 14.39±11.60 | 10.27±11.53 | 1.86 | 0.07 |
| IOG.R | 11.81±2.96 | 10.89±4.14 | 1.35 | 0.18 |  | 0.24±0.02 | 0.23±0.04 | 3.05 | 0.004 |  | 17.98±15.80 | 23.21±24.81 | -1.33 | 0.19 |
| FFG.R | 13.39±2.51 | 11.53±3.70 | 3.10 | 0.002 |  | 0.25±0.02 | 0.24±0.03 | 3.09 | 0.003 |  | 29.4±23.58 | 31.07±23.76 | -0.37 | 0.71 |
| PHG.L | 10.32±3.47 | 8.62±4.37 | 2.25 | 0.03 |  | 0.23±0.03 | 0.21±0.06 | 2.78 | 0.006 |  | 20.93±16.36 | 15.51±13.64 | 1.86 | 0.07 |
| AMYG.L | 10.62±3.78 | 8.06±4.16 | 3.37 | 0.001 |  | 0.23±0.05 | 0.20±0.05 | 2.89 | 0.005 |  | 24.86±17.97 | 19.96±18.78 | 1.39 | 0.17 |
| AMYG.R | 10.05±3.72 | 7.51±3.69 | 3.57 | 0.001 |  | 0.23±0.04 | 0.19±0.05 | 3.48 | 0.001 |  | 26.14±30.19 | 15.02±15.22 | 2.37 | 0.02 |

Data was represented as mean ± standard deviation

Abbreviations: HC, healthy control; PD, Parkinson’s disease; HOFC, high-order functional connectivity; L, left; R, right; ROL, rolandic operculum; SMA, supplementary motor area; PCG, posterior cingulate gyrus; PHG, parahippocampal gyrus; AMYG, amygdala; CAL, calcarine fissure; LING, lingual gyrus; IOG, inferior occipital gyrus; FFG, fusiform gyrus; PoCG, postcentral gyrus; SMG, supramarginal gyrus; ANG, angular gyrus; PCUN, precuneus; PUT, lenticular nucleus, putamen; THA, thalamus; HES, heschl gyrus

Supplementary Table 3. Nodal topological metrics of LOFC-networks for each group

| Brain region | Nodal degree | | | |  | Nodal efficiency | | | |  | Nodal betweenness | | | |
| --- | --- | --- | --- | --- | --- | --- | --- | --- | --- | --- | --- | --- | --- | --- |
|  | HC | PD | T | *P* |  | HC | PD | T | *P* |  | HC | PD | T | *P* |
| ANG.R | 7.28±3.47 | 10.20±3.77 | -4.20 | <0.001 |  | 0.21±0.04 | 0.24±0.03 | -3.76 | <0.001 |  | 7.77±7.17 | 9.99±8.99 | -1.43 | 0.16 |
| PCG.R | 6.17±3.80 | 9.04±4.35 | -3.68 | <0.001 |  | 0.20±0.05 | 0.22±0.04 | -3.09 | 0.003 |  | 5.52±6.12 | 7.30±8.25 | -1.29 | 0.20 |
| CAL.R | 10.81±3.51 | 12.62±3.80 | -2.58 | 0.01 |  | 0.24±0.03 | 0.25±0.03 | -2.33 | 0.02 |  | 9.03±7.97 | 14.63±13.08 | -2.74 | 0.007 |
| THA.R | 8.40±5.29 | 12.57±4.50 | -4.39 | <0.001 |  | 0.21±0.07 | 0.25±0.03 | -4.12 | <0.001 |  | 14.28±18.03 | 22.96±16.78 | -2.59 | 0.01 |
| THA.L | 8.79±5.35 | 12.92±4.38 | -4.36 | <0.001 |  | 0.21±0.07 | 0.26±0.03 | -4.04 | <0.001 |  | 17.47±16.20 | 26.21±21.27 | -2.43 | 0.02 |
| ROL.R | 10.70±3.78 | 8.97±4.19 | 2.27 | 0.03 |  | 0.24±0.03 | 0.22±0.04 | 2.66 | 0.009 |  | 17.33±14.23 | 18.03±20.58 | -0.21 | 0.84 |
| ROL.L | 11.01±3.89 | 9.10±3.19 | 2.76 | 0.007 |  | 0.24±0.03 | 0.23±0.03 | 2.96 | 0.004 |  | 19.04±15.51 | 18.72±15.50 | 0.11 | 0.91 |
| HES.L | 6.49±4.32 | 5.20±3.66 | 1.67 | 0.10 |  | 0.20±0.05 | 0.18±0.06 | 2.90 | 0.004 |  | 6.35±8.61 | 4.25±6.06 | 1.45 | 0.15 |
| FFG.R | 15.24±3.47 | 13.54±3.44 | 2.56 | 0.01 |  | 0.27±0.02 | 0.26±0.02 | 2.71 | 0.008 |  | 31.82±21.27 | 30.55±21.33 | 0.31 | 0.76 |
| AMYG.R | 8.11±4.75 | 5.61±3.70 | 3.03 | 0.003 |  | 0.21±0.06 | 0.18±0.06 | 2.69 | 0.008 |  | 12.92±14.64 | 8.65±8.23 | 1.83 | 0.07 |
| PHG.L | 8.66±4.13 | 6.58±4.08 | 2.63 | 0.01 |  | 0.23±0.04 | 0.20±0.06 | 2.91 | 0.004 |  | 13.11±14.36 | 7.13±7.76 | 2.64 | 0.01 |
| AMYG.L | 9.58±5.04 | 5.79±4.12 | 4.24 | <0.001 |  | 0.23±0.06 | 0.19±0.06 | 3.31 | 0.001 |  | 19.88±20.86 | 7.75±9.18 | 3.81 | <0.001 |

Data was represented as mean ± standard deviation

Abbreviations: HC, healthy control; PD, Parkinson’s disease; HOFC, high-order functional connectivity; L, left; R, right; ROL, rolandic operculum; PCG, posterior cingulate gyrus; PHG, parahippocampal gyrus; AMYG, amygdala; CAL, calcarine fissure; FFG, fusiform gyrus; ANG, angular gyrus; THA, thalamus; HES, heschl gyrus

Supplementary Table 4. Modular topological metrics of HOFC-networks and LOFC-networks for each group

| Metric | HOFC | | | |  | LOFC | | | |
| --- | --- | --- | --- | --- | --- | --- | --- | --- | --- |
|  | HC | PD | T | *P* |  | HC | PD | T | *P* |
| SMN-DMN | 0.44±0.17 | 0.39±0.17 | 1.38 | 0.17 |  | 0.45±0.15 | 0.40±0.15 | 1.56 | 0.12 |
| SMN-FPN | 0.26±0.11 | 0.27±0.12 | -0.32 | 0.75 |  | 0.26±0.10 | 0.26±0.10 | -0.13 | 0.89 |
| SMN-VN | 0.32±0.15 | 0.29±0.15 | 1.14 | 0.26 |  | 0.31±0.12 | 0.29±0.13 | 0.95 | 0.35 |
| SMN-SN | 0.34±0.12 | 0.28±0.12 | 2.20 | 0.03 |  | 0.26±0.08 | 0.24±0.09 | 1.33 | 0.19 |
| DMN-FPN | 0.44±0.14 | 0.50±0.17 | -2.20 | 0.03 |  | 0.45±0.10 | 0.49±0.13 | -2.16 | 0.03 |
| DMN-VN | 0.42±0.20 | 0.43±0.17 | -0.55 | 0.58 |  | 0.43±0.16 | 0.46±0.14 | -1.07 | 0.29 |
| DMN-SN | 0.40±0.15 | 0.41±0.13 | -0.59 | 0.55 |  | 0.34±0.12 | 0.35±0.11 | -0.43 | 0.67 |
| FPN-VN | 0.15±0.08 | 0.19±0.10 | -2.27 | 0.03 |  | 0.17±0.08 | 0.20±0.09 | -1.90 | 0.06 |
| FPN-SN | 0.16±0.08 | 0.16±0.08 | -0.28 | 0.78 |  | 0.13±0.06 | 0.14±0.05 | -0.51 | 0.61 |
| VN-SN | 0.12±0.07 | 0.17±0.10 | -3.09 | 0.002 |  | 0.18±0.06 | 0.16±0.08 | -2.71 | 0.008 |
| SMN | 0.43±0.10 | 0.39±0.12 | 2.16 | 0.03 |  | 0.40±0.09 | 0.35±0.10 | 2.34 | 0.02 |
| DMN | 0.66±0.17 | 0.69±0.20 | -0.94 | 0.35 |  | 0.65±0.15 | 0.67±0.18 | -0.70 | 0.47 |
| FPN | 0.22±0.06 | 0.20±0.06 | 1.31 | 0.19 |  | 0.22±0.05 | 0.21±0.05 | 1.29 | 0.20 |
| VN | 0.30±0.05 | 0.26±0.06 | 3.07 | 0.003 |  | 0.29±0.04 | 0.27±0.05 | 2.67 | 0.009 |
| SN | 0.18±0.05 | 0.16±0.05 | 1.64 | 0.10 |  | 0.16±0.05 | 0.14±0.05 | 1.97 | 0.05 |

Data was represented as mean ± standard deviation

Abbreviations: HC, healthy control; PD, Parkinson’s disease; HOFC, high-order functional connectivity; LOFC, low-order functional connectivity; SMN, sensorimotor network; DMN, default mode network; FPN, fronto-parietal network; VN, visual network; SN, subcortical network

Supplementary Table 5. Classification performances of HOFC-networks and LOFC-networks using a functionally defined atlas

|  | AUC |  | Accuracy (%) | Sensitivity (%) | Specificity (%) |
| --- | --- | --- | --- | --- | --- |
| HOFC | 0.76 |  | 67.57 | 58.82 | 75.00 |
| LOFC | 0.70 |  | 66.67 | 62.75 | 70.00 |

Abbreviations: HOFC, high-order functional connectivity; LOFC, low-order functional connectivity; AUC, area under the curve

Supplementary Table 6. Global topological metrics of HOFC-networks and LOFC-networks for each group using a functionally defined atlas

| Metric | HOFC | | | |  | LOFC | | | |
| --- | --- | --- | --- | --- | --- | --- | --- | --- | --- |
|  | HC | PD | T | *P* |  | HC | PD | T | *P* |
| γ | 0.76±0.10 | 0.72±0.11 | 2.40 | 0.02 |  | 0.61±0.07 | 0.60±0.07 | 1.03 | 0.31 |
| λ | 0.43±0.01 | 0.43±0.01 | 0.60 | 0.55 |  | 0.41±0.01 | 0.41±0.01 | 0.32 | 0.75 |
| σ | 0.70±0.09 | 0.66±0.10 | 2.12 | 0.04 |  | 0.59±0.07 | 0.58±0.07 | 0.92 | 0.36 |
| Lp | 0.68±0.03 | 0.68±0.03 | -0.07 | 0.94 |  | 0.65±0.02 | 0.65±0.02 | -0.002 | 0.99 |
| Cp | 0.27±0.01 | 0.27±0.01 | 0.81 | 0.42 |  | 0.25±0.01 | 0.24±0.01 | 1.80 | 0.74 |
| Eglob | 0.25±0.03 | 0.24±0.01 | 2.17 | 0.03 |  | 0.25±0.01 | 0.25±0.01 | -0.13 | 0.90 |
| Eloc | 0.33±0.01 | 0.33±0.01 | 1.43 | 0.16 |  | 0.32±0.01 | 0.32±0.01 | 1.49 | 0.14 |

Data was represented as mean ± standard deviation

Abbreviations: HC, healthy control; PD, Parkinson’s disease; HOFC, high-order functional connectivity; LOFC, low-order functional connectivity; γ, normalized clustering coefficient; λ, normalized characteristic path length; σ, small-worldness; Lp, characteristic path length; Cp, clustering coefficient; Eglob, global efﬁciency; Eloc, local efﬁciency

Supplementary Table 7. Nodal topological metrics that specific to HOFC-networks for each group using a functionally defined atlas

| Brain region | Nodal degree | | | |  | Nodal efficiency | | | |  | Nodal betweenness | | | |
| --- | --- | --- | --- | --- | --- | --- | --- | --- | --- | --- | --- | --- | --- | --- |
|  | HC | PD | T | *P* |  | HC | PD | T | *P* |  | HC | PD | T | *P* |
| FPN-Cing1_R | 42.73±19.62 | 54.03±18.55 | -3.07 | 0.003 |  | 0.22±0.04 | 0.24±0.02 | -3.45 | 0.001 |  | 88.14±87.50 | 80.26±44.35 | 0.58 | 0.56 |
| FPN-Par1_L | 45.05±14.80 | 55.84±19.46 | -3.28 | 0.001 |  | 0.23±0.02 | 0.24±0.03 | -2.97 | 0.004 |  | 95.86±73.74 | 73.97±45.66 | 1.82 | 0.07 |
| FPN-Par3_R | 43.34±15.72 | 54.79±18.22 | -3.52 | 0.001 |  | 0.23±0.02 | 0.24±0.02 | -3.55 | 0.001 |  | 80.24±48.21 | 79.26±71.03 | 0.09 | 0.93 |
| FPN-PFCl11_R | 43.55±13.36 | 52.98±18.10 | -3.12 | 0.002 |  | 0.23±0.02 | 0.24±0.03 | -3.12 | 0.002 |  | 93.92±48.79 | 76.55±51.09 | 1.81 | 0.07 |
| FPN-PFCl12_R | 48.96±17.62 | 60.51±16.37 | -3.52 | 0.001 |  | 0.24±0.03 | 0.25±0.02 | -3.3 | 0.001 |  | 98.35±72.99 | 85.90±46.05 | 1.04 | 0.30 |
| FPN-PFCl14_R | 47.94±16.47 | 60.27±16.36 | -3.91 | <0.001 |  | 0.23±0.02 | 0.25±0.02 | -3.86 | <0.001 |  | 94.23±59.35 | 107.56±86.15 | -0.95 | 0.34 |
| FPN-PFCl8_L | 43.77±13.98 | 53.57±16.53 | -3.36 | 0.001 |  | 0.23±0.02 | 0.24±0.02 | -3.06 | 0.003 |  | 82.30±59.91 | 73.13±50.92 | 0.85 | 0.40 |
| FPN-PFCl8_R | 45.69±17.19 | 56.75±16.05 | -3.45 | 0.001 |  | 0.23±0.03 | 0.25±0.02 | -3.28 | 0.001 |  | 85.76±45.12 | 79.08±53.06 | 0.71 | 0.48 |
| FPN-PFCmp1_L | 45.97±17.11 | 56.11±16.01 | -3.18 | 0.002 |  | 0.23±0.02 | 0.24±0.02 | -3.01 | 0.003 |  | 90.19±54.99 | 86.70±62.24 | 0.31 | 0.76 |
| DMN-pCunPCC2_R | 46.68±18.16 | 60.08±18.31 | -3.82 | <0.001 |  | 0.23±0.03 | 0.25±0.02 | -3.82 | <0.001 |  | 84.74±64.51 | 84.83±52.09 | -0.01 | 0.99 |
| DMN-pCunPCC4_L | 48.21±19.19 | 58.52±17.14 | -2.94 | 0.004 |  | 0.23±0.03 | 0.25±0.02 | -3.31 | 0.001 |  | 92.53±81.08 | 93.28±66.88 | -0.05 | 0.96 |
| DMN-pCunPCC8_L | 51.00±16.46 | 60.65±17.86 | -2.94 | 0.004 |  | 0.24±0.03 | 0.25±0.02 | -2.91 | 0.004 |  | 101.41±72.9 | 104.44±87.64 | -0.20 | 0.84 |
| DMN-PFC16_L | 45.58±15.96 | 54.53±16.07 | -2.91 | 0.004 |  | 0.23±0.03 | 0.24±0.03 | -2.48 | 0.015 |  | 68.27±47.28 | 62.23±48.63 | 0.66 | 0.51 |
| DAN-Post19_R | 65.57±14.34 | 56.7±14.64 | 3.19 | 0.002 |  | 0.26±0.02 | 0.25±0.02 | 2.83 | 0.006 |  | 66.31±52.44 | 78.23±67.91 | -1.03 | 0.30 |
| LN-TempPole1_L | 64.33±15.02 | 54.53±20.16 | 2.90 | 0.005 |  | 0.26±0.02 | 0.24±0.03 | 2.78 | 0.007 |  | 122.2±76.75 | 109.48±69.13 | 0.90 | 0.37 |
| LN-TempPole1_R | 58.75±18.76 | 47.86±17.95 | 3.08 | 0.003 |  | 0.25±0.04 | 0.23±0.03 | 1.85 | 0.067 |  | 98.77±64.17 | 90.65±53.65 | 0.71 | 0.48 |
| LN-TempPole4_L | 41.57±20.24 | 53.44±20.05 | -3.07 | 0.003 |  | 0.22±0.04 | 0.24±0.04 | -2.64 | 0.009 |  | 64.69±73.07 | 64.47±49.35 | 0.02 | 0.99 |
| SMN-1_L | 57.67±18.52 | 47.05±19.35 | 2.92 | 0.004 |  | 0.25±0.03 | 0.23±0.03 | 2.28 | 0.025 |  | 98.13±91.42 | 90.03±74.50 | 0.50 | 0.62 |
| SMN-10_R | 66.08±17.67 | 52.99±21.25 | 3.51 | 0.001 |  | 0.26±0.02 | 0.24±0.03 | 3.48 | 0.001 |  | 118.39±107.48 | 86.35±64.61 | 1.85 | 0.07 |
| SMN-11_L | 68.73±17.43 | 56.85±16.11 | 3.67 | <0.001 |  | 0.26±0.02 | 0.25±0.02 | 3.37 | 0.001 |  | 88.66±49.84 | 91.19±82.32 | -0.20 | 0.84 |
| SMN-12_R | 64.53±16.21 | 54.20±19.72 | 3.00 | 0.003 |  | 0.26±0.02 | 0.24±0.03 | 2.92 | 0.004 |  | 88.12±58.74 | 106.28±70.85 | -1.46 | 0.15 |
| SMN-15_R | 55.99±20.80 | 42.45±20.54 | 3.40 | 0.001 |  | 0.24±0.03 | 0.22±0.05 | 3.06 | 0.003 |  | 57.17±45.02 | 57.42±51.18 | -0.03 | 0.98 |
| SMN-19_R | 63.89±16.41 | 53.5±19.46 | 3.02 | 0.003 |  | 0.25±0.02 | 0.24±0.03 | 3.02 | 0.003 |  | 60.15±39.03 | 74.26±48.75 | -1.68 | 0.10 |
| SMN-4_L | 63.19±17.19 | 51.56±22.53 | 3.05 | 0.003 |  | 0.25±0.03 | 0.24±0.04 | 2.88 | 0.005 |  | 91.60±61.94 | 95.66±88.35 | -0.28 | 0.78 |
| SN-CAU-body-R | 43.41±19.52 | 55.32±18.25 | -3.27 | 0.001 |  | 0.23±0.03 | 0.25±0.02 | -3.50 | 0.001 |  | 85.07±59.20 | 88.18±57.13 | -0.28 | 0.78 |
| SN-CAU-DA-R | 46.13±16.88 | 56.17±18.71 | -2.95 | 0.004 |  | 0.23±0.03 | 0.25±0.03 | -2.83 | 0.006 |  | 99.73±92.12 | 83.17±35.78 | 1.20 | 0.23 |
| SN-CAU-VA-R | 44.87±18.96 | 59.48±17.10 | -4.19 | <0.001 |  | 0.23±0.03 | 0.25±0.02 | -4.13 | <0.001 |  | 94.11±71.59 | 108.65±64.82 | -1.10 | 0.27 |
| SN-lAMY-R | 56.02±21.22 | 43.86±20.18 | 3.05 | 0.003 |  | 0.24±0.03 | 0.23±0.04 | 2.48 | 0.015 |  | 108.06±69.35 | 82.52±54.73 | 2.11 | 0.04 |
| SN-mAMY-R | 56.71±21.36 | 41.97±20.03 | 3.69 | <0.001 |  | 0.24±0.04 | 0.22±0.04 | 2.99 | 0.003 |  | 91.41±61.96 | 73.14±57.08 | 1.59 | 0.11 |
| SN-PUT-DA-L | 43.47±16.33 | 54.54±17.78 | -3.39 | 0.001 |  | 0.23±0.03 | 0.24±0.03 | -2.68 | 0.008 |  | 85.09±72.06 | 85.66±53.55 | -0.05 | 0.96 |
| SN-THA-DAm-L | 41.12±18.66 | 53.31±19.05 | -3.37 | 0.001 |  | 0.22±0.03 | 0.24±0.03 | -3.81 | <0.001 |  | 64.74±48.66 | 100.93±92.55 | -2.61 | 0.01 |
| SN-THA-DAm-R | 41.37±17.97 | 53.50±20.08 | -3.33 | 0.001 |  | 0.22±0.03 | 0.24±0.03 | -3.76 | <0.001 |  | 74.03±79.80 | 103.99±119.17 | -1.56 | 0.12 |
| SN-THA-VAip-L | 46.82±21.23 | 61.11±15.87 | -3.92 | <0.001 |  | 0.23±0.04 | 0.25±0.02 | -4.08 | <0.001 |  | 89.91±61.40 | 136.23±96.94 | -3.02 | 0.003 |
| SN-THA-VAip-R | 51.92±19.40 | 65.34±17.64 | -3.75 | <0.001 |  | 0.24±0.03 | 0.26±0.02 | -4.00 | <0.001 |  | 110.80±78.81 | 135.87±83.08 | -1.61 | 0.11 |
| VN-10_L | 60.73±15.48 | 50.88±18.87 | 2.99 | 0.003 |  | 0.25±0.02 | 0.24±0.03 | 2.51 | 0.013 |  | 83.68±99.61 | 85.22±57.62 | -0.10 | 0.92 |
| VN-14_L | 61.37±17.61 | 50.19±17.74 | 3.29 | 0.001 |  | 0.25±0.02 | 0.24±0.03 | 2.86 | 0.005 |  | 74.36±53.64 | 79.30±46.39 | -0.51 | 0.61 |
| VN-3_R | 68.75±16.08 | 57.29±16.18 | 3.70 | <0.001 |  | 0.26±0.02 | 0.25±0.02 | 2.96 | 0.004 |  | 115.56±79.72 | 112.91±85.21 | 0.17 | 0.87 |
| VN-8_L | 71.89±15.56 | 62.84±16.76 | 2.92 | 0.004 |  | 0.27±0.02 | 0.26±0.02 | 2.51 | 0.014 |  | 104.44±58.62 | 121.51±70.78 | -1.38 | 0.17 |

Data was represented as mean ± standard deviation

Abbreviations: HC, healthy control; PD, Parkinson’s disease; HOFC, high-order functional connectivity; L, left; R, right; FPN, fronto-parietal network; DMN, default mode network; DAN, dorsal attention network; LN, limbic network; SMN, sensorimotor network; SN, subcortical network; VN, visual network; Cing, cingulate; Par, parietal; PFC, prefrontal cortex; pCunPCC, precuneus posterior cingulate cortex; post, post central; TempPole, temporal pole; CAU, caudate; DA, dorsoanterior; VA, ventroanterior; AMY, amygdala; PUT, putamen; THA, thalamus; DAm, medial dorsoanterior; VAip, posterior division;

Supplementary Table 8. Modular topological metrics of HOFC-networks and LOFC-networks for each group using a functionally defined atlas

| Metric | HOFC | | | |  | LOFC | | | |
| --- | --- | --- | --- | --- | --- | --- | --- | --- | --- |
|  | HC | PD | T | *P* |  | HC | PD | T | *P* |
| DAN-DMN | 0.30±0.09 | 0.36±0.11 | -2.86 | 0.01 |  | 0.35±0.08 | 0.39±0.11 | -2.39 | 0.02 |
| DAN-FPN | 0.32±0.09 | 0.33±0.08 | -0.65 | 0.52 |  | 0.35±0.07 | 0.36±0.08 | -0.36 | 0.72 |
| DAN-LN | 0.12±0.04 | 0.12±0.04 | 0.52 | 0.60 |  | 0.11±0.04 | 0.11±0.04 | -0.07 | 0.94 |
| DAN-SN | 0.21±0.09 | 0.23±0.07 | -1.65 | 0.10 |  | 0.21±0.07 | 0.22±0.06 | -1.22 | 0.23 |
| DAN-VAN | 0.31±0.11 | 0.31±0.10 | 0.22 | 0.83 |  | 0.31±0.09 | 0.30±0.08 | 0.36 | 0.72 |
| DMN-SN | 0.50±0.17 | 0.58±0.19 | -2.58 | 0.01 |  | 0.46±0.14 | 0.52±0.15 | -2.46 | 0.02 |
| FPN-DMN | 0.56±0.15 | 0.65±0.21 | -2.84 | 0.01 |  | 0.59±0.12 | 0.66±0.17 | -2.76 | 0.01 |
| FPN-SN | 0.25±0.08 | 0.29±0.10 | -2.62 | 0.01 |  | 0.22±0.06 | 0.26±0.07 | -2.92 | 0.004 |
| LN-DMN | 0.30±0.09 | 0.32±0.11 | -0.83 | 0.41 |  | 0.27±0.08 | 0.29±0.09 | -1.08 | 0.28 |
| LN-FPN | 0.13±0.04 | 0.15±0.05 | -1.65 | 0.10 |  | 0.12±0.04 | 0.13±0.04 | -1.94 | 0.06 |
| LN-SN | 0.16±0.04 | 0.17±0.05 | -1.54 | 0.13 |  | 0.13±0.04 | 0.14±0.05 | -1.29 | 0.20 |
| SMN-DAN | 0.56±0.20 | 0.54±0.17 | 0.59 | 0.56 |  | 0.54±0.16 | 0.52±0.13 | 0.71 | 0.48 |
| SMN-DMN | 0.50±0.22 | 0.49±0.19 | 0.24 | 0.81 |  | 0.56±0.21 | 0.55±0.17 | 0.25 | 0.80 |
| SMN-FPN | 0.24±0.11 | 0.32±0.12 | -3.37 | 0.001 |  | 0.27±0.10 | 0.32±0.10 | -2.83 | 0.01 |
| SMN-LN | 0.21±0.09 | 0.17±0.06 | 2.41 | 0.02 |  | 0.19±0.07 | 0.17±0.06 | 1.3 | 0.20 |
| SMN-SN | 0.45±0.15 | 0.45±0.15 | 0.05 | 0.96 |  | 0.42±0.11 | 0.42±0.11 | -0.13 | 0.90 |
| SMN-VAN | 0.60±0.15 | 0.61±0.17 | -0.45 | 0.66 |  | 0.57±0.11 | 0.57±0.13 | -0.19 | 0.85 |
| VAN-DMN | 0.30±0.10 | 0.28±0.12 | 0.55 | 0.58 |  | 0.34±0.10 | 0.32±0.11 | 0.87 | 0.39 |
| VAN-FPN | 0.26±0.08 | 0.25±0.07 | 1.09 | 0.28 |  | 0.27±0.07 | 0.26±0.07 | 0.88 | 0.38 |
| VAN-LN | 0.10±0.04 | 0.09±0.03 | 2.15 | 0.03 |  | 0.09±0.03 | 0.09±0.03 | 1.01 | 0.31 |
| VAN-SN | 0.27±0.07 | 0.25±0.09 | 1.47 | 0.14 |  | 0.25±0.06 | 0.24±0.07 | 0.81 | 0.42 |
| VN-DAN | 0.39±0.13 | 0.35±0.11 | 1.62 | 0.11 |  | 0.39±0.09 | 0.37±0.09 | 2.38 | 0.12 |
| VN-DMN | 0.58±0.22 | 0.59±0.21 | -0.02 | 0.98 |  | 0.61±0.19 | 0.63±0.18 | -0.44 | 0.66 |
| VN-FPN | 0.23±0.09 | 0.29±0.10 | -3.08 | 0.003 |  | 0.26±0.09 | 0.31±0.09 | -2.54 | 0.01 |
| VN-LN | 0.17±0.06 | 0.16±0.06 | 0.28 | 0.78 |  | 0.15±0.05 | 0.16±0.06 | -0.71 | 0.48 |
| VN-SN | 0.30±0.10 | 0.34±0.10 | -2.24 | 0.03 |  | 0.29±0.09 | 0.33±0.09 | -2.11 | 0.04 |
| VN-SMN | 0.75±0.28 | 0.60±0.23 | 3.16 | 0.002 |  | 0.72±0.20 | 0.61±0.19 | 3.11 | 0.002 |
| VN-VAN | 0.25±0.10 | 0.26±0.10 | -0.13 | 0.90 |  | 0.27±0.08 | 0.26±0.09 | 0.11 | 0.91 |
| DAN | 0.24±0.05 | 0.22±0.05 | 3.18 | 0.002 |  | 0.26±0.04 | 0.23±0.04 | 3.93 | <0.001 |
| DMN | 0.78±0.21 | 0.83±0.22 | -1.24 | 0.22 |  | 0.80±0.17 | 0.84±0.18 | -1.27 | 0.21 |
| FPN | 0.24±0.05 | 0.24±0.06 | -0.07 | 0.94 |  | 0.26±0.04 | 0.26±0.05 | 0.04 | 0.97 |
| LN | 0.06±0.02 | 0.05±0.02 | 1.70 | 0.09 |  | 0.05±0.01 | 0.05±0.02 | 1.23 | 0.22 |
| SN | 0.25±0.07 | 0.24±0.05 | 0.55 | 0.58 |  | 0.24±0.06 | 0.23±0.06 | 1.11 | 0.27 |
| SMN | 0.77±0.16 | 0.61±0.16 | 5.09 | <0.001 |  | 0.72±0.14 | 0.59±0.13 | 5.04 | <0.001 |
| VAN | 0.23±0.07 | 0.23±0.06 | 0.76 | 0.45 |  | 0.23±0.05 | 0.22±0.05 | 1.23 | 0.22 |
| VN | 0.43±0.08 | 0.37±0.08 | 4.12 | <0.001 |  | 0.44±0.06 | 0.39±0.07 | 3.74 | <0.001 |

Data was represented as mean ± standard deviation

Abbreviations: HC, healthy control; PD, Parkinson’s disease; HOFC, high-order functional connectivity; LOFC, low-order functional connectivity; DAN, dorsal attention network; DMN, default mode network; FPN, fronto-parietal network; LN, limbic network; SN, subcortical network; VAN, ventral attention network; SMN, sensorimotor network; VN, visual network
